# Evaluation of MC-80 automatic blood cell morphology analyzer in identifying the morphology of blood cells in patients with hematological diseases and normal samples

**DOI:** 10.1097/MD.0000000000043323

**Published:** 2025-07-18

**Authors:** Jing-Jing Chen, Hui-Hui Zhou, Fei Sun, ZhaoFei Wang, Ling Ding, Fangfang Yang, WanRong Shi

**Affiliations:** a Department of Clinical Laboratory, The First Affiliated Hospital of Anhui Medical University, Hefei, Anhui Province, China; b Department of Clinical Laboratory, Anhui Public Health Clinical Center, Hefei, Anhui Province, China; c Health Examination Center, The First Affiliated Hospital of Anhui Medical University, Hefei, Anhui Province, China; d Health Examination Center, Anhui Public Health Clinical Center, Hefei, Anhui Province, China.

**Keywords:** blood cell morphology analyzer, hematological diseases, laboratory automation, manual microscopic examination

## Abstract

**Background:**

This study evaluates the performance of the MC-80 automatic blood cell morphology analyzer in identifying the morphology of blood cells in patients with hematological diseases and normal samples.

**Methods:**

A total of 320 patients with hematological diseases and 200 healthy persons with health examination from the health examination center between January 2023 and May 2024 were selected as research subjects, and 520 venous blood samples were collected in this study. The morphology of white blood cells, nucleated red blood cells, platelets, and mature red blood cells was all detected by the MC-80 automatic blood cell morphology analyzer and compared with the manual microscopic examination. The ability of the MC-80 automatic blood cell morphology analyzer in identifying the morphology of blood cells in patients with hematological diseases and normal samples was evaluated.

**Results:**

MC-80 demonstrated high precision, sensitivity, specificity, negative predictive value, and positive predictive value indiscriminating different forms of blood cells. There was no significant difference in the accuracy of platelet clumps and segmented neutrophils preclassified by MC-80 compared with manual verification. The accuracy of all the cells was >90% after verification. Turnaround time showed no significant difference in total turnaround time between MC-80 analysis and manual counting.

**Conclusion:**

The MC-80 automatic blood cell morphology analyzer is a reliable and powerful blood cell morphology analyzer in analyzing the morphology of most blood cells in patients with hematological diseases and normal samples.

## 1. Introduction

Complete blood counts detected by conventional automated hematology analyzers with 5 classifications are an essential and important detection method, especially for patients with hematological diseases.^[1,2]^ Therefore, manual microscopic examination of peripheral blood cell smears is still an essential method in clinical diagnosis and treatment of hematological diseases and related diseases.^[3,4]^ That is because manual microscopy has a core value in identifying abnormal cells, verifying instrument results, and detecting specific pathogens. Clinically, it is necessary to combine the results of automated instruments and manual microscopy to ensure the comprehensiveness and accuracy of diagnosis.

MC-80 (Mindray, Shenzhen, China) used in our laboratory for morphological analysis is a novel automatic blood cell morphology analyzer. MC-80 based on traditional microscopic image analysis technology combined with measuring technology is a new type of automatic blood cell morphology analysis system. After staining, the blood smears were placed under the oil lens and scanned at low magnification (100×). In a few minutes, clear images of blood cells were captured and distinguished at high magnification (1000×). In this way, MC-80 can automatically detect the morphology of individual blood cells on peripheral blood smears through a neural network and store clear images of identified cells in a large database. It not only has the ability to classify white blood cells (WBCs) but also provides the morphological information of red blood cells (RBCs) and platelets. Compared with traditional microscopy, it has many advantages, such as improving work efficiency, reducing work intensity, and providing assistance from morphological experts to small- or medium-volume clinical laboratories by means of telemedicine. In the past few years, there have been some scientific studies focusing on the performance of an automatic blood cell morphology analyzer in identifying the morphology of blood cells.^[5,6]^ However, the evaluation indicators of MC-80 in detecting all kinds of blood cells from patients with hematological diseases and normal samples are not complete. In this study, we also evaluated the performance of the MC-80 blood cell morphology analyzer by means of comparing MC-80 preclassification and verification with manual microscopic counting and calculating the sample turnaround time (TAT) of MC-80 analysis and manual microscopic counting, respectively, to evaluate work efficiency.

## 2. Materials and methods

### 2.1. Study subjects

This study was conducted from January 2023 to May 2024 in the Clinical Laboratory of The First Affiliated Hospital of Anhui Medical University. We obtained 520 venous blood samples from 320 patients with hematological diseases and 200 healthy persons. Table 1 summarizes the characteristics of all the study subjects enrolled in our study. This research protocol was approved by the Ethics Committee of The First Affiliated Hospital of Anhui Medical University, and informed consent was not required because there was no need to collect additional blood samples.

**Table 1:** Characteristics of study subjects.

Sample characteristics	Total (N = 520)
Age (yr), median (IQR)	46 (33–72)
Male, n (%)	282 (54.23%)
Diagnosis, n (%)	
Acute myeloid leukemia	88 (16.92%)
Multiple myeloma	54 (10.38%)
Lymphoma	44 (8.46%)
Aplastic anemia	34 (6.54%)
Myelodysplastic syndromes	20 (3.85%)
Acute lymphoblastic leukemia	18 (3.46%)
Chronic myelogenous leukemia	38 (7.31%)
Chronic lymphocytic leukemia	12 (2.31%)
Primary myelofibrosis	2 (0.38%)
Polycythemia vera	10 (1.92%)
Normal healthy subjects	200 (38.46%)

IQR = interquartile range.

### 2.2. Sample collection

Venous blood samples from all the patients were collected in vacuum tubes containing K_2_-EDTA anticoagulant and measured with the Mindray BC6800 automatic hematology analyzer. After that, peripheral blood smears were automatically prepared by the Mindray automatic Stainer SC-120 and dyed with Wright-Giemsa staining solution.

The staining steps were as follows: first, all the slides were stained by Wright-Giemsa staining solution without methanol prefixing for about 2 minutes; second, phosphate buffer was added to these slides for about 6 minutes; after that, the slides were rinsed 4× with water; and finally, these slides were dried for about 5 minutes. The stained peripheral blood cell smears were placed in a slide basket and were ready for testing.

### 2.3. MC-80 detection and verification

The MC-80 automatic blood cell morphology analyzer is an automatic blood cell morphology identification instrument that uses an automated microscope and a digital camera. White blood cells can be preclassified into band neutrophils, segmented neutrophils, eosinophils, basophils, lymphocytes, monocytes, reactive lymphocytes, abnormal lymphocytes, abnormal promyelocytes, immature granulocytes, and blast cells. Immature granulocytes include promyelocytes, myelocytes, and metamyelocytes.

RBCs can be preclassified into nucleated RBCs (NRBCs) and mature RBCs. The MC-80 system can count >10 kinds of RBCs in real time, classify and identify the morphology of RBCs, automatically evaluate the morphology and size of RBCs, and quantitatively analyze the morphological changes of RBCs. In addition, the preclassification results of giant platelets and platelet clumps can also be acquired using MC-80. This system can perform real-time quantitative analysis on the number of platelets, platelet morphology, and platelet clumps (light, medium, and heavy).

Every day after turning up the MC-80, the internal quality control was carried out first. After internal quality control passes, the test samples were analyzed in strict accordance with the standard operating procedures of the instrument. In the preclassification of blood cells, nearly 200 WBCs were collected from each slide.^[7,8]^ In fact, >200 cells were collected per slide because platelets and red cells were also included in addition to the WBCs mentioned above.

Furthermore, it was important for us to note that when the number of WBCs in a blood sample was <2.0 × 10^9^/L, 2 blood smears from this sample were prepared to meet the need of leukocyte classification. There are 32 samples that had <2×10^9^/L WBCs in total. These spare blood smears were to be used for classification and analysis in order to increase the preclassification number of WBCs to 200.

In total, 552 blood smears were preclassified by the MC-80 system, 2 experienced technicians were selected to review the preclassified results, while 2 senior morphological technicians were selected to manually count cells with a microscope.^[9]^ These morphological microscopy technologists were required to have an intermediate or above professional title, a morphological examination score of≥80, morphological microscopy experience of>5 years, and a comparison coincidence rate of≥80% to perform the examination and report the results.

### 2.4. Manual microscopic counting

In addition to MC-80 analyzer preclassification and verification, manual microscopic examination was performed based on the Clinical and Laboratory Standards Institute (CLSI) H20-A2 guidelines.^[7]^ According to the international microscopic review rules,^[10]^ all the same blood smears were analyzed by using the “double-blind method,” and 200 WBCs were counted by 2 technical supervisors, respectively, with rich working experience. First, search for the field of view under low magnification (100×) with the light microscope, and then, classify and count cells according to cell morphological characteristics under high magnification (1000×). Furthermore, the morphology of RBCs and platelets was also examined at the same time. The counting results of different cells by 2 morphological microscopy technologists were averaged for statistical analysis. Besides that, the number of platelets was estimated in the whole blood smear, and platelet aggregation, parasites, and abnormal cells were also observed.

### 2.5. Evaluation of work efficiency by comparing TAT

Work efficiency was also evaluated by comparing TAT between the MC-80 analysis and manual microscopic counting. Steps A and a referred to the completion of the preparation of the slides, including Wright-Giemsa staining, rinsing, and drying. It should be noted that steps A and a were different. Step A meant that blood smears were automatically prepared, stained, and rinsed by the Mindray automatic Stainer SC-120; Step a meant that blood smears were prepared, stained, and rinsed all by manual operation. Steps B and b involved placing the slide under the microscope lens and selecting the appropriate area for microscopy. Steps C and c referred to MC-80 preclassification and manual microscopic counting, respectively. Furthermore, step D referred to verification, and step d referred to recording the results.

### 2.6. Statistical analysis

SPSS 27.0 software (International Business Machines Corp., [IBM], Armonk) was used for statistical analysis. Measurement data are presented as $\bar{x}\pm s$, and the differences between the 2 groups were compared by the Student *t* test or the Mann-Whitney *U* test. The rates in the 2 groups were compared using the χ^2^ test. The Passing-Bablok regression, Pearson correlation, and Bland-Altman plot were used to describe the correlation of preclassification, verification, and manual counting. Pearson correlation coefficients (r) were interpreted as follows: <0.30, negligible; 0.30 to 0.50, low; 0.50 to 0.70, moderate; 0.70 to 0.90, high; and 0.90 to 1.00, very high. Sensitivity, specificity, negative predictive value, and positive predictive value were used to evaluate the performance of MC-80 in discriminating abnormal blood cells after verification. Differences were statistically significant when the *P* value was <.05.

## 3. Result

### 3.1. Preclassification of blood smears by the MC-80 system

A total of 103,574 images from these blood smears were scanned and recorded by the MC-80 system, including normal cells and abnormal cells. Band neutrophils, segmented neutrophils, eosinophils, basophils, lymphocytes, and monocytes are normal cells. Blasts, immature granulocytes, reactive lymphocytes, plasma cells, abnormal lymphocytes, abnormal promyelocytes, NRBCs, giant platelets, and platelet clumps are abnormal cells. There were 740 unidentified cells, 3832 band neutrophils, 50,624 segmented neutrophils, 3568 eosinophils, 1040 basophils, 28,288 lymphocytes, 3944 monocytes, 500 blasts, 524 immature granulocytes, 268 reactive lymphocytes, 10 plasma cells, 5 abnormal lymphocytes, 7 abnormal promyelocytes, 3448 NRBCs, 3056 giant platelet, 3088 platelet clumps, and 632 smudge cells. The interface of the MC-80 system is shown in Figure S1, Supplemental Digital Content, https://links.lww.com/MD/P398.

### 3.2. Manual verification analysis of blood cells after preclassification

When all the blood smear samples were preclassified by the MC-80 system, a total of 740 cells were unclassified, and the unrecognized rate was 0.71%. The unidentified cells were manually rechecked and reclassified. Among them, 88 (11.89%) were segmented neutrophils, 36 (4.86%) were monocytes, 4 (0.54%) were blasts, 40 (5.41%) were immature granulocytes, 64 (8.65%) were NRBCs, and 388 (52.43%) were smudge cells. The number of smudge cells accounted for >50%. Among them, 21 eosinophils and 16 monocytes were misclassified as lymphocytes and smudge cells in blood smears from normal healthy subjects. Fifty-two blasts misclassified were all from patients with hematological diseases. Forty-four blasts were misclassified into 8 segmented neutrophils, 12 eosinophils, 4 basophils, 4 immature granulocytes, 4 giant platelets, and 12 platelet clumps. In addition, 4 blasts were unidentified. They were all from patients with acute myeloid leukemia and chronic myelogenous leukemia. The results after manual verification are shown in Table 2.

**Table 2:** Blood samples were preclassified by the MC-80 system and compared with the results of manual verification.

Cell class	Preclassification			Verification										
	Total cell count	wrong cell count		Band neutrophils	Segmented neutrophils			Lymphocytes		Monocytes	Eosinophils		Basophils	
Unidentified	740	740		24	88			20		36	4		20	
Band neutrophils	3832	496		0	236			88		52	0		32	
Segmented neutrophils	50,624	1212		620	0			296		60	48		72	
Lymphocytes	28,288	244		8	12			0		0	8		0	
Monocytes	3944	156		24	8			4		0	8		8	
Eosinophils	3568	560		36	4			148		112	0		80	
Basophils	1040	32		0	4			8		0	0		0	
Blasts	500	52		4	0			0		8	0		0	
Immature granulocyte	524	84		16	16			12		0	16		8	
Plasma cells	10	2		0	0			0		0	0		0	
Reactive lymphocytes	268	60		0	0			40		4	0		0	
Abnormal lymphocytes	5	0		0	0			0		0	0		0	
Abnormal Promyelocytes	7	1		0	0			0		1	0		0	
Nucleated RBCs	3448	348		12	164			68		0	0		0	
Giant platelet	3056	24		0	0			4		0	0		0	
Platelet clumps	3088	36		4	0			4		0	0		0	
Smudge cells	632	16		4	0			0		0	12		0	
Total amount	103,574	4063		752	532			792		373	116		220	
Cell class	Verification													
	Blasts	Immature granulocyte	Reactive lymphocytes			Plasma cells	Nucleated RBCs		Giant platelet			Platelet clumps		Smudge cells
Unidentified	4	40	12			0	64		28			12		388
Band neutrophils	0	4	0			0	4		8			0		72
Segmented neutrophils	8	28	20			0	0		24			8		28
Lymphocytes	0	0	12			0	0		0			8		196
Monocytes	0	8	8			0	0		4			0		84
Eosinophils	12	60	0			0	0		16			0		92
Basophils	4	0	0			0	0		0			0		16
Blasts	0	12	4			0	0		0			0		24
Immature granulocyte	4	0	8			0	0		0			0		4
Plasma cells	0	0	2			0	0		0			0		0
Reactive lymphocytes	0	4	0			0	0		0			0		12
Abnormal lymphocytes	0	0	0			0	0		0			0		0
Abnormal Promyelocytes	0	0	0			0	0		0			0		0
Nucleated RBCs	0	4	0			0	0		40			0		60
Giant platelet	4	0	0			0	0		0			12		4
Platelet clumps	12	0	8			0	0		8			0		0
Smudge cells	0	0	0			0	0		0			0		0
Total amount	48	160	94			0	68		128			40		980

RBC = red blood cell.

### 3.3. Evaluation of the accuracy of the MC-80 system in identifying different cells

The accuracy of the MC-80 system in identifying different cells was evaluated by comparing the preclassification results and verification results with manual microscopic examination, which is regarded as the gold standard. The results showed that there was no significant difference in the accuracy of segmented neutrophils and platelet clumps preclassified by MC-80 compared with manual verification (*P* > .05). However, the accuracy of band neutrophils, lymphocytes, monocytes, eosinophils, basophils, blasts, immature granulocytes, reactive lymphocytes, NRBCs, and giant platelet preclassified by MC-80 was significantly less than manual verification (*P* < .05; Table 3).

**Table 3:** Evaluation of the accuracy of the MC-80 system by comparing preclassification and manual verification with microscopy.

Cell class	Number of cells			Percentage of correctly classified cells (%)			
	Preclassification	Verification	Manual counting	Preclassification versus manual counting	Verification versus manual counting	χ²	*P* value
Band neutrophils	3832	4088	4168	80.04%	98.08%	35.482	.000
Segmented neutrophils	50,624	49,944	54,084	91.36%	92.35%	4.512	.09
Lymphocytes	28,288	28,836	31,184	89.93%	92.47%	29.251	.000
Monocytes	3944	4160	4532	73.81%	91.79%	16.790	.000
Eosinophils	3568	3224	3260	90.61%	98.90%	8.482	.000
Basophils	1040	1228	1290	78.14%	95.19%	35.180	.000
Blasts	500	496	506	88.54%	98.02%	32.133	.000
Immature granulocytes	524	600	652	67.48%	92.02%	30.294	.000
Reactive lymphocytes	268	302	324	64.19%	93.20%	32.507	.000
Nucleated RBCs	3448	3168	3265	94.95%	97.03%	25.105	.000
Giant platelet	3056	3260	3408	88.97%	95.66%	18.054	.000
Platelet clumps	3088	3092	3280	93.05%	94.27%	5.106	.08

RBC = red blood cell.

### 3.4. Correlation analysis of the results of preclassification, verification, and manual microscopic examination

In this study, correlation analysis was used to describe the correlation of preclassification, verification, and manual counting. The results showed that there were high correlations of >0.90 between preclassification versus verification and preclassification versus manual counting in segmented neutrophils, band neutrophils, lymphocytes, eosinophils, blasts, NRBCs, giant platelets, and platelet clumps, while moderate correlations were between 0.70 and 0.90 or low correlations were between 0.50 and 0.70 in basophils, reactive lymphocytes, and immature granulocytes (*P* < .001). In addition, high correlations of >0.90 were found between verification and manual counting in all cells (Figure S2, Supplemental Digital Content, https://links.lww.com/MD/P400).

### 3.5. Evaluation of the precision of the MC-80 system in identifying different cells

The precision of the MC-80 system in identifying different cells was evaluated using repeatability.^[11]^ In this study, 10 samples including 5 normal samples and 5 abnormal samples were randomly selected, and each sample was detected 5× a day over 5 consecutive days. The average number of cells on each slide was calculated. We observed that NRBCs had the highest coefficients of variation (CV) %, while a giant platelet had the lowest CV%. In addition, the CV% of band neutrophils, segmented neutrophils, lymphocytes, monocytes, eosinophils, basophils, blasts, reactive lymphocytes, giant platelets, and platelet clumps were <0.7%, with 0.69%, 0.08%, 0.05%, 0.20%, 0.21%, 0.18%, 0.25%, 0.11%, 0.05%, and 0.12%, respectively. However, the CV% of NRBCs and immature granulocytes was 3.78% and 3.63%, respectively (Table 4).

**Table 4:** Precision of MC-80 preclassification (n = 250).

Cell class	Mean number of cells per slide	Repeatability	
	Mean	SD	CV%
Band neutrophils	1.85	1.28	0.69
Segmented neutrophils	157.70	1.23	0.08
Lymphocytes	24.95	1.42	0.05
Monocytes	4.2	0.84	0.20
Eosinophils	1.5	0.31	0.21
Basophils	1.0	0.40	0.18
Blasts	5.45	1.37	0.25
Immature granulocytes	6.80	4.13	3.63
Reactive lymphocytes	4.54	0.83	0.11
Nucleated RBCs	5.60	4.12	3.78
Giant platelet	2.75	0.83	0.05
Platelet clumps	15.90	3.43	0.12

CV = coefficients of variation, RBC = red blood cell, SD = standard deviation.

### 3.6. Evaluation of the performance of MC-80 in discriminating different blood cells after verification

**Figure 1.**
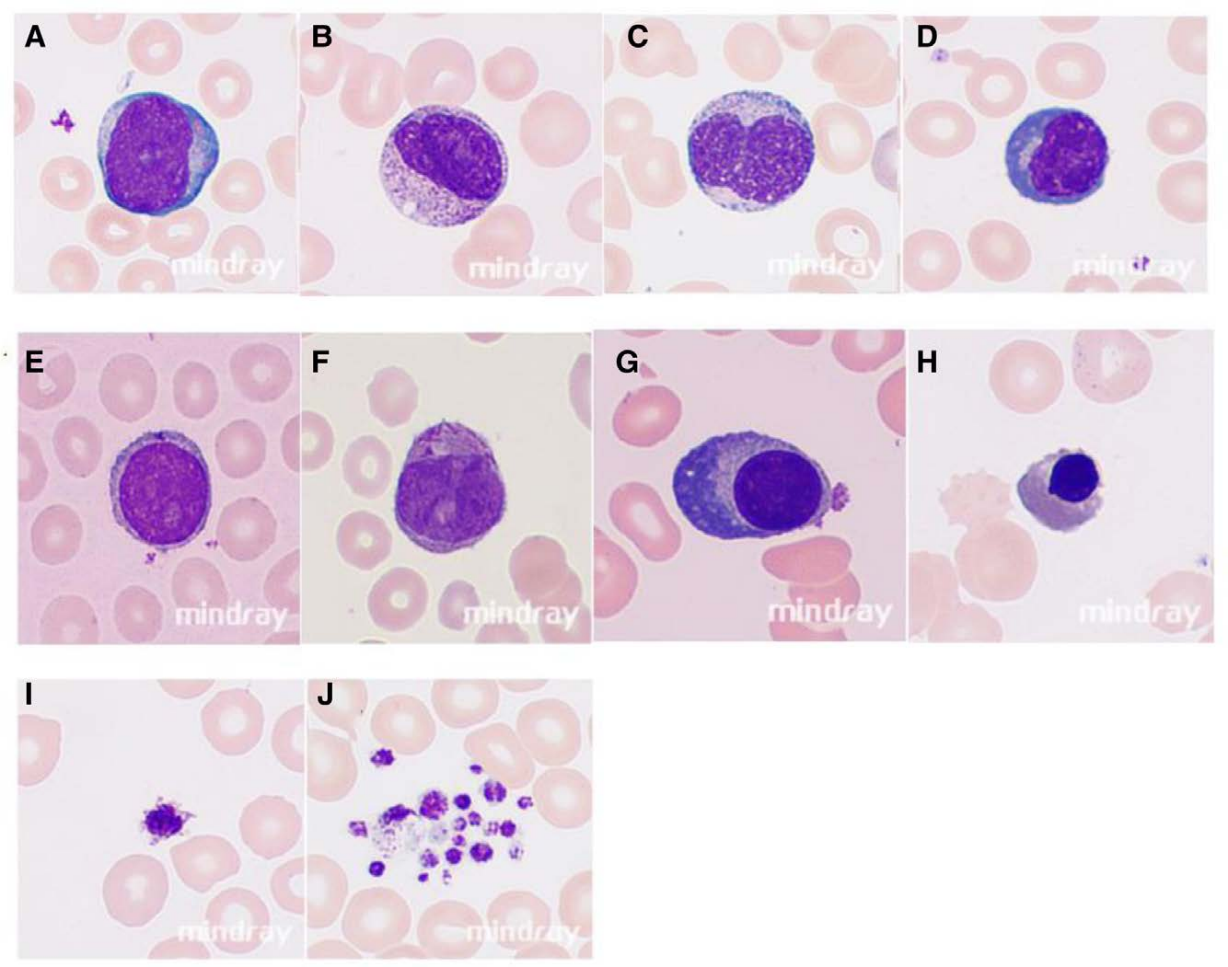
Abnormal morphology of blood cells detected by MC-80. (A) Blasts. (B and C) Immature granulocytes. (D) Reactive lymphocytes. (E) Abnormal lymphocytes. (F) Abnormal promyelocytes. (G) Plasma cells. (H) Nucleated red blood cells. (I) Giant platelets. (J) Platelet aggregation.

Abnormal white cells including blasts, promyelocytes, myelocytes, metamyelocytes, and reactive lymphocytes were captured and analyzed by the MC-80 system. NRBCs, giant platelets, and platelet clumps were also analyzed (Table S1, Supplemental Digital Content, https://links.lww.com/MD/P396). We observed that most sensitivity values exceeded 90%, except for reactive lymphocytes (68.87%); specificity values were all above or very close to 99%; NPV was >98% in all cases except for NRBCs (89.91%); and PPV also showed good, though more variable, results (ranging from 77.61% for reactive lymphocytes to 99.21% for NRBCs). In addition to that, abnormal cells detected by MC-80 are shown in Figure 1.

### 3.7. Comparison of TAT between MC-80 and manual microscopic examination

We could observe that TAT of manual microscopic counting was significantly longer than that of preclassification (*P* < .05), but TAT of verification was significantly longer than that of recording the results (*P* < .05). Thus, we found that there was no significant difference in total TAT between MC-80 analysis and manual microscopic counting (*P* > .05; Table 5).

**Table 5:** Comparison of TAT between MC-80 and manual microscopic examination.

TAT, sec ($\bar{x}\pm \;S$) (n = 520)					
Process	MC-80	Process	Manual examination	*t*	*P* value
Step A	534.09 ± 11.29	Step a	559.24 ± 10.02	4.629	<.05
Step B	121.3 ± 7.97	Step b	107.4 ± 8.08	3.87	<.05
Step C	384.8 ± 8.92	Step c	478.7 ± 7.65	25.28	<.05
Step D	149.8 ± 8.07	Step d	67.2 ± 12.30	17.76	<.05
Total TAT	1060.50 ± 16.32	Total TAT	1069.1 ± 28.79	1.30	>.05

TAT = turnaround time.

## 4. Discussion

Nowadays, the routine blood test is playing an important role in auxiliary diagnosis, differential diagnosis, treatment, and prognosis of diseases.^[12,13]^ However, information about the morphology characterization of WBCs, RBCs, and platelets is limited in a routine blood examination.^[14,15]^ In fact, with the increasing number of clinical specimens, manual microscopic morphological examination of peripheral blood cell smears requires a lot of time and well-trained morphological technicians.^[16]^ Thus, it is more and more difficult to achieve the quantity and quality of manual microscopic examination according to the requirements of the reexamination rules, especially in clinical laboratories of large hospitals. For this reason, with the continuous development of science and technology, a large number of automatic blood cell morphology image analysis instruments have been developed and put into use in recent years.^[17–19]^

The MC-80 system is an automatic digital image analysis system, which can automatically analyze blood smears to meet the needs of clinical laboratories. It can extract different parameters of morphological features of WBCs, RBCs, and platelets for preclassification and recognition. Various WBCs and non-WBCs such as RBCs and platelets can also be precharacterized by an MC-80 analyzer. Previous study by Zini et al^[20]^ assessed the performance of the Mindray MC-80 for hematology patients with frequent leukemic and dysplastic cells. They found an excellent correlation between the test and the reference method for normal and abnormal circulating cell types, except for reactive lymphocytes. Ye et al^[21]^ collected only 100 abnormal peripheral blood smears to compare the leukocyte differential performance of Mindray MC-80 with Sysmex DI-60. They also found the great performance of MC-80 in accurately preclassifying blasts in patients, exhibiting high sensitivity (92.0%), which was in line with our study. Merino et al^[22]^ found that the MC-80 shows high performance for WBC differentials for both normal samples and patients with haematological diseases compared with DM9600. In this research, the performance of MC-80 in identifying different cells from 320 patients with hematological diseases and 200 healthy persons was comprehensively analyzed. A total of 103,574 cells were scanned and preclassified by the MC-80 system. In addition, giant platelets and platelet clumps were also analyzed.

The results showed that the accuracy of most cells preclassified by MC-80 was >80% except basophils, immature granulocytes, and reactive lymphocytes. This was similar to the scientific literature written by Khongjaroensakun et al,^[23]^ with the overall preclassification accuracy ranging from 82% to 92%. Similar results were observed by Zini et al^[20]^ who found that the pass rate of distributional inaccuracy was >80% for pathological cells (except 75.1% for reactive lymphocytes). In addition, we also found that the accuracy of all the cells was >90% after verification using manual microscopic counting as a reference. The results also showed that the accuracy of platelet clumps preclassified by MC-80 was >90%, and there was no significant difference compared with manual verification. Therefore, it can be a great method to save time and improve work efficiency. The platelet clump results of the routine blood test can help us make a conclusion. Platelet clumps or giant platelets can cause pseudothrombocytopenia (PTCP). PTCP refers to the phenomenon that platelets accumulate in external anticoagulant blood, which causes the blood analyzer to be unable to identify platelets, and the platelet count result is significantly lower than the true value of platelets. PTCP can occur in autoimmune diseases, chronic inflammation, viral or bacterial infections, metabolic syndrome, tumors, and treatment with certain drugs. PTCP can also occur in healthy individuals. A previous study by Woo et al^[24]^ found that the platelet counts determined using the DI-60 were not sufficiently accurate to be accepted. This is contrary to what we found with MC-80, which is worth our further study and discussion.

Correlation analysis demonstrated that there were high correlations between preclassification versus verification and preclassification versus manual counting in segmented neutrophils, band neutrophils, lymphocytes, monocytes, eosinophils, blasts, NRBCs, giant platelets, and platelet clumps, while there were moderate correlations or low correlations in basophils, reactive lymphocytes, and immature granulocytes. Manual counting versus verification showed high correlation in all cells, probably because the number of basophils, reactive lymphocytes, and immature granulocytes was very small, and the results would be greatly affected by the area, which was chosen to read on the slide. This is consistent with the article written by Ye et al^[21]^ who found that both DI-60 and MC-80 exhibited a very high correlation with the manual counting results in neutrophils, lymphocytes, and blasts.

Evaluation of the precision of the MC-80 system demonstrated that MC-80 showed high precision in identifying band neutrophils, segmented neutrophils, lymphocytes, monocytes, eosinophils, basophils, blasts, reactive lymphocytes, plasma cells, platelet clumps, and giant platelets, and low precision in NRBCs and immature granulocytes. The CV% of NRBCs and immature granulocytes was 3.78% and 3.63%, respectively, which represented the low precision. The low precision may be due to the complex morphology of these cells^[25,26]^ and the morphological changes of the cells caused by the increased placement time of slides, which may lead to the misjudgment of the instrument. Similar results were observed by Zini et al.^[20]^ However, they only measured the standard deviation and CV of 2 abnormal samples, and they did not analyze a sufficient population of healthy subjects to obtain reference values.

MC-80 also demonstrated high sensitivity, specificity, negative predictive value, and positive predictive value in discriminating abnormal cells after verification, except reactive lymphocytes. Once again, this result proved that we should pay more attention to the results of manual counting for abnormal cells with complex morphology, especially reactive lymphocytes. Reactive lymphocytes are generally categorized into 3 main types based on their morphological features in peripheral blood smears, such as plasma cell type, monocyte type, and immature type. However, due to the complexity of reactive lymphocyte morphology, it is easy to be misidentified. In this study, we observed that 208 reactive lymphocytes were correctly classified and 60 reactive lymphocytes misclassified were from 37 patients with hematological diseases, which do not usually directly lead to the increase of reactive lymphocytosis but may occur in coinfection, inflammation, or other complications. Among them, 12 patients were acute myeloid leukemia, 5 patients were multiple myeloma, 4 patients were lymphoma, 6 patients were multiple myeloma, and 10 patients were myelodysplastic syndromes. Thus, we should pay more attention to the identification of reactive lymphocytes in these diseases.

Lee et al^[6]^ thought that DC-1, which was also a digital morphology analyzer, might create unnecessary workload because the TAT of DC-1 was not ideal. In the analysis of TAT between MC-80 and manual microscopic examination, we found that the difference between the total TAT of MC-80 analysis and manual microscopic counting was not statistically significant. If the performance of MC-80 in identifying different cells was the same as manual microscopic examination, this is undoubtedly an effective way to help us save time, reduce workload, and improve work efficiency on the whole, especially in large laboratories.

There were several limitations in our study. First, a relatively small number of blood samples were analyzed. Second, these blood samples were all from our hospital, and we need to collect more samples from different hospitals. Finally, some cells were not analyzed in our study due to the low number of patients with certain diagnoses. In conclusion, the MC-80 system is a useful tool to analyze blood smears of patients with hematological diseases and normal samples. Although manual microscopic examination is required for identifying the morphology of some cells such as basophils, reactive lymphocytes, and immature granulocytes, we believe that with the increasing number of samples and the continuous expansion of the built-in sample library, the performance of MC-80 in identifying various cells will be continuously improved, and it will have a broad prospect in clinical application in the future.

## Author contributions

**Investigation:** Jing-Jing Chen, Hui-Hui Zhou, WanRong Shi.

**Project administration:** Jing-Jing Chen, Fei Sun.

**Writing – review & editing:** Jing-Jing Chen, Fangfang Yang.

**Resources:** Hui-Hui Zhou, Fangfang Yang.

**Supervision:** Hui-Hui Zhou, Ling Ding.

**Conceptualization:** ZhaoFei Wang.

**Data curation:** ZhaoFei Wang.

**Software:** Ling Ding.

**Funding acquisition:** WanRong Shi.

## Supplementary Material


