# Supplementary material for: Evaluation of MC-80 automatic blood cell morphology analyzer in identifying the morphology of blood cells in patients with hematological diseases and normal samples
Source: Medicine (Baltimore). 2025 Jul 18;104(29):e43323. doi: 10.1097/MD.0000000000043323 (PMC12282804; doi:10.1097/MD.0000000000043323)
Supplement: Supplementary file 1 [file medi-104-e43323-s001.docx]

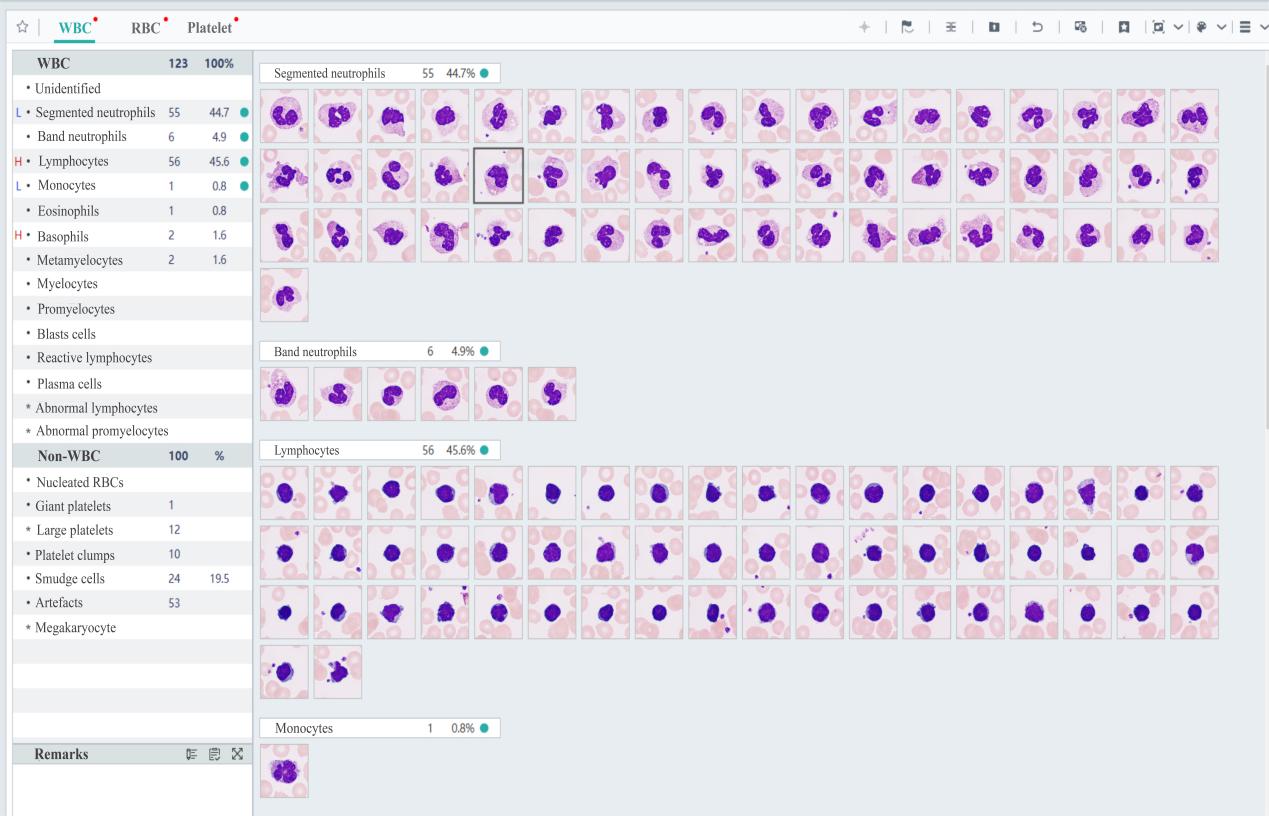


**Figure S1 Interface of** **MC-80 automatic blood cell morphology classification and recognition system**

Abbreviations: WBC, white blood cells; RBC, red blood cells
