# Supplementary material for: Evaluation of MC-80 automatic blood cell morphology analyzer in identifying the morphology of blood cells in patients with hematological diseases and normal samples
Source: Medicine (Baltimore). 2025 Jul 18;104(29):e43323. doi: 10.1097/MD.0000000000043323 (PMC12282804; doi:10.1097/MD.0000000000043323)
Supplement: Supplementary file 2 [file medi-104-e43323-s002.docx]

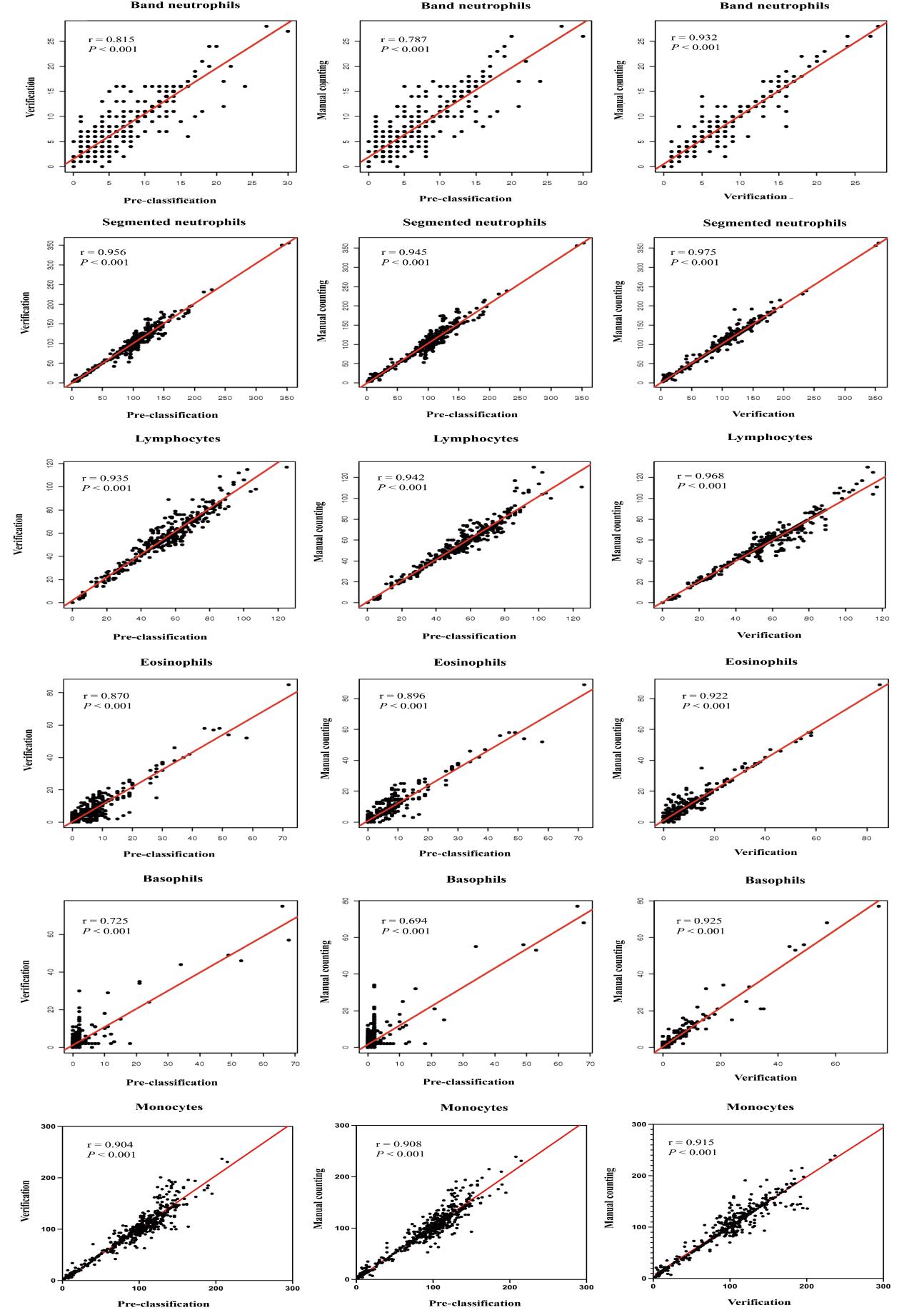


**Figure S2A Correlation analysis of pre-classification, verification, and manual microscopic examination(bands, segmented neutrophils, lymphocytes, eosinophils, basophils, and monocytes).**


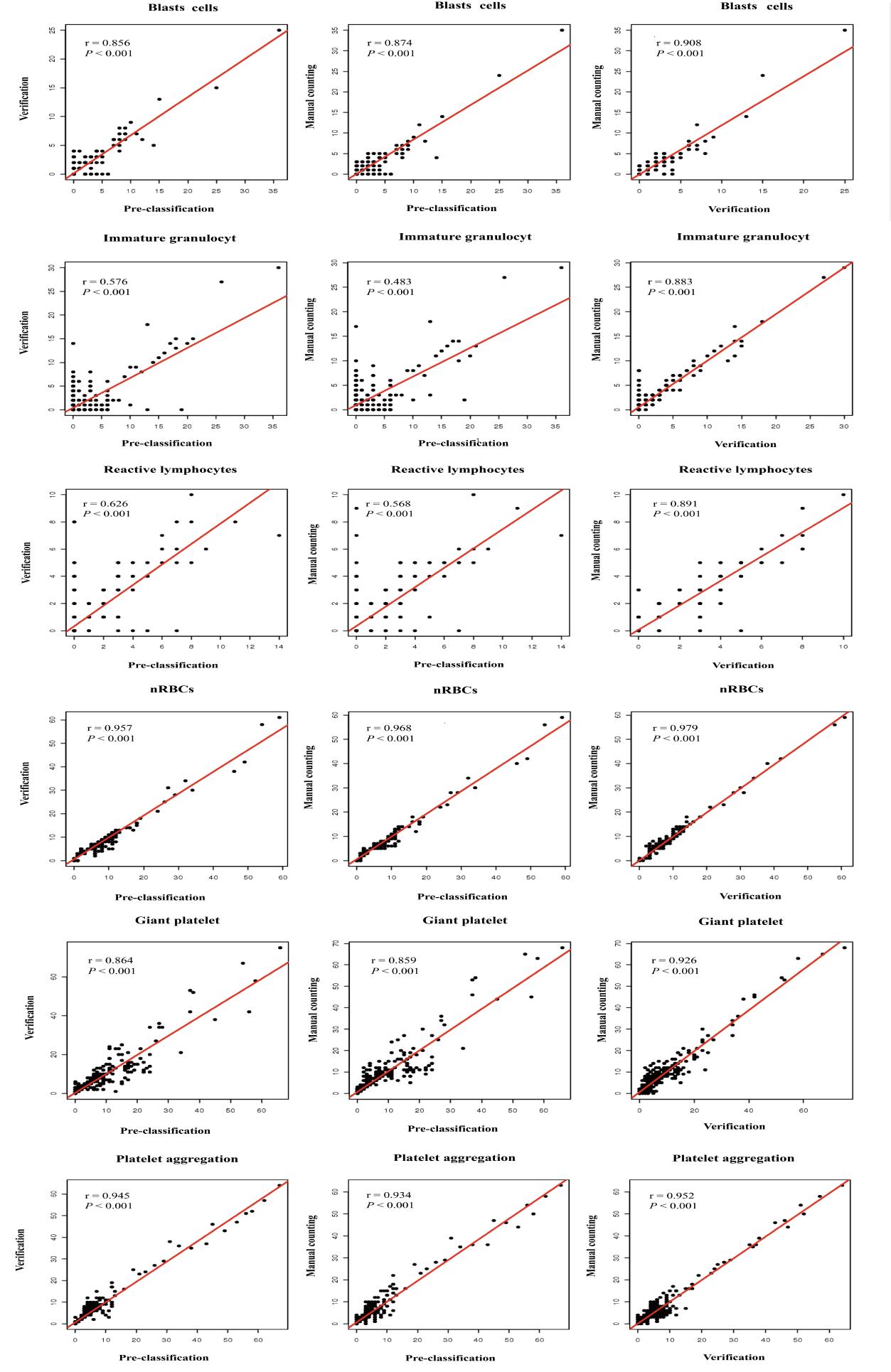


**Figure S2B Correlation analysis of pre-classification, verification, and manual microscopic examination(the remaining cells).**
