# Supplementary material for: Evaluation of MC-80 automatic blood cell morphology analyzer in identifying the morphology of blood cells in patients with hematological diseases and normal samples
Source: Medicine (Baltimore). 2025 Jul 18;104(29):e43323. doi: 10.1097/MD.0000000000043323 (PMC12282804; doi:10.1097/MD.0000000000043323)
Supplement: Supplementary file 3 [file medi-104-e43323-s003.docx]

**Table S1 Ability to discriminate abnormal blood cells after verification**

| Abnormal morphology | Sensitivity | Specificity | Negative predictive value | Positive  predictive value |
| --- | --- | --- | --- | --- |
| Blasts | 90.32 | 99.85 | 99.95 | 89.60 |
| Promyelocytes | 90.10 | 98.14 | 99.58 | 91.46 |
| Myelocytes | 90.65 | 97.42 | 99.25 | 83.31 |
| Metamyelocytes | 93.50 | 98.33 | 98.42 | 89.64 |
| Reactive lymphocytes | 68.87 | 99.94 | 99.91 | 77.61 |
| Nucleated RBCs | 97.85 | 99.17 | 89.91 | 99.21 |
| Giant platelets | 96.68 | 99.07 | 99.12 | 94.65 |
| Platelet clumps | 98.71 | 99.31 | 99.07 | 98.52 |
